# Anti-telomerase T cells adoptive transfer

**DOI:** 10.18632/aging.101336

**Published:** 2017-11-30

**Authors:** Francesco De Sanctis, Rosalinda Trovato, Stefano Ugel

**Affiliations:** Ageing and Alzheimers Institute and ANZAC Research Institute, University of Sydney and Concord Hospital, Sydney, Australia

**Keywords:** cancer immunotherapy, adoptive cell transfer (ACT), telomerase (TERT), TCR-redirected T-cells, tumor

**Figure 1:**
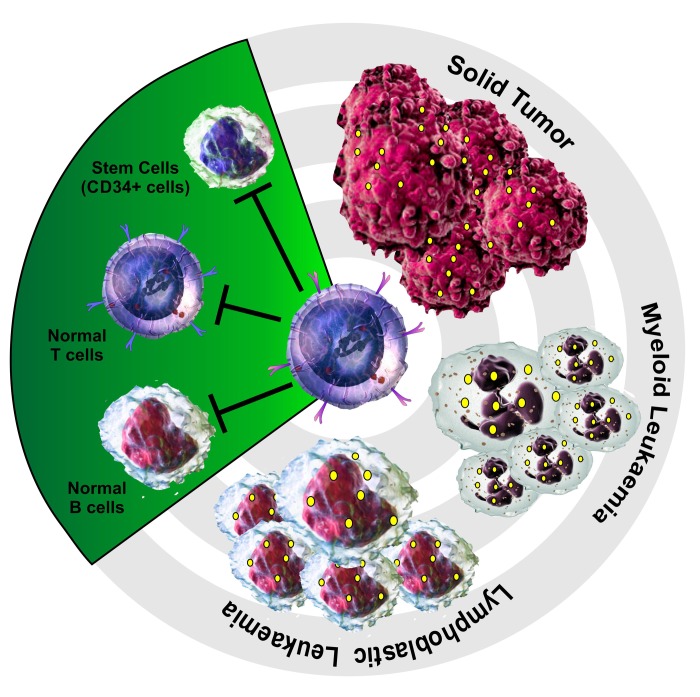
TERT-based adoptive cell therapy is an universal cancer immunotherapeutic approach hTERT_865-873_-specific, TCR-engineered T-cells, as golden bullets, are able to selectively eliminate antigen positive (yellow spots) specific targets such as lymphoblastic and myeloid leukaemia cells and different solid tumor cells without affecting normal B and T lymphocytes as well as stem cells.

The concept of adoptive cell therapy (ACT) was born more than 70 years ago with the purpose to ready supply immune-associated weapons for fighting lethal diseases, such as cancer. This strategy overcomes the limitations of cancer vaccines based on developing a tumor specific immune response in immune compromised tumor bearing hosts in which a tolerance against tumor antigens has been already established. The first clinical trial exploiting T cell-based ACT was performed in 1988 on melanoma patients: tumor infiltrating T lymphocytes (TILs) were isolated from melanoma tumors and infused in the same patients after *ex vivo* expansion. Since that, many advances were reached. Today the most challenging issues limiting the efficacy of patient-derived tumor specific T cell adoptive transfer relied both on the terminal differentiation status of transferred T cells and on their low affinity binding to tumor associated antigens (TAA), which are proteins expressed in healthy tissues even if at a lower level. The elimination of most reactive clones targeting self-proteins is indeed the consequence of central tolerance action on T cell repertoire. DNA engineering techniques circumvented this issue. Transgenic T cell receptors (TCRs) or chimeric antigen receptors (CARs) conferred the ability of recognizing TAA with higher affinity and eventually without MHC restriction, unleashing thus all the cytotoxic arsenal of T cells against tumor. The higher chances to target self-tissues, which raise when central tolerance is by-passed, represents the most important concern. Thus the choice of the TAA and of the TCR avidity is crucial to reach the greatest efficacy on tumor with low risk of off-targets and consequent side effects. In this context, telomerase reverse transcriptase (TERT), a protein that extends chromosomal ends (telomeres) preventing genomic instability and allowing indefinite cell proliferation potential, represents an ideal candidate. Indeed, TERT is generally expressed at very low levels in healthy tissues characterized by high self-renewal abilities (such as bone marrow and testes) whereas it is reactivated in cancer cells. In fact, TERT activity is observed in about 85% of human tumors of various histological types qualifying this protein as a universal tumor antigen (UTA) [1]. By using a DNA vaccine targeting mouse TERT, we recently isolated a polyclonal cytotoxic T lymphocytes (CTLs) population recognizing mTERT_198-205_ in H2-K^b^ context able to restrict growth of different types of tumors after their *in vivo* infusion with very low side effects [2]. To prove the translating potential of our findings we mirrored the experiment set up in a human setting: after vaccinating HLA-A*0201 transgenic mice using human TERT encoding DNA, we identified *in vitro* several clones able to recognize a large number of cancer cell lines [2]. Moreover, we validated the ability of high affinity CTLs to *in vivo* control human cancer progression of both immortalized cancer cells and, also, patient-derived HLA-matched cancer stem cells [2]. Therefore, we decided to clone the sequences of the α and β chains of the anti-telomerase TCR into a retroviral vector able to transduce naïve T cells [3]. ACT of hTERT_865–873_ specific TCR-engineered human T cells (PCT/IB2016/051510) was able to control the *in vivo* progression of different human malignancies: human chronic lymphocytic leukemia (B-CLL) human B-cell acute lymphoblastic leukaemia (B-ALL), acute myeloid leukaemia (AML) and different solid cancers [3, 4]. Finally, our side effects studies on human immune reconstituted (HIR) mice showed very limited toxicity against mature granulocytes but not toward human hematopoietic progenitors [3]. Thus, the flexibility of usage on both solid and liquid tumors of this magic bullet, together with minimal safety concerns, qualify hTERT_865–873_ specific TCR-engineered human T cell ACT as a new precision weapon for cancer immune therapy (Figure 1). Nonetheless the cutting edge preclinical results, there is still space to improve the efficacy of TERT-specific CTL based ACT. Tumor progression is coupled with the development of an immune suppressive microenvironment hostile to T cell homing and function: the induction of a more favorable milieu for T lymphocytes represents thus a critical requirement for enhancing T cell fitness and action on tumor cells. In this context, we recently designed and positively tested a drug able to block the *in vivo* generation of peroxynitrites and to improve consequent-ly transferred-T cell infiltration and persistence at the tumor site [5]. We also dissected the role of complement system activation on tumor endothelium in orchestrating T cell homing to tumor after ACT [6]; as well as, we identified a specific subset of dendritic cells, named TipDC, characterized as inducible nitric oxide synthase (iNOS) and tumor necrosis factor α (TNFα) producers, able to improve TERT-based ACT efficacy when injected in the tumor-microenvironment [7]. In the next future, we plan to combine ACT with immune checkpoint inhibitors (anti-PD1, anti-PD-L1) to enhance T cells proliferation, survival and cytotoxic activity with the final aim of providing a long lasting immune therapy efficacy and protection against tumor recur-rence. In conclusion, we believe that combination immunotherapy based on anti-telomerase T cells adop-tive transfer together with immunomodulators will open a new frontier for cancer immunotherapy that will elevate the quality standards of patient care and will result in better survival and lower side effects.
